# Dynamic Causal Modelling of Active Vision

**DOI:** 10.1523/JNEUROSCI.2459-18.2019

**Published:** 2019-08-07

**Authors:** Thomas Parr, M. Berk Mirza, Hayriye Cagnan, Karl J. Friston

**Affiliations:** ^1^Wellcome Centre for Human Neuroimaging, Institute of Neurology, University College London, WC1N 3BG, United Kingdom,; ^2^MRC Brain Network Dynamics Unit at the University of Oxford, Oxford OX1 3TH, United Kingdom, and; ^3^Nuffield Department of Clinical Neurosciences, University of Oxford, Oxford OX3 9DU, United Kingdom

**Keywords:** active vision, attention, dynamic causal modelling, eye-tracking, magnetoencephalography, visual neglect

## Abstract

In this paper, we draw from recent theoretical work on active perception, which suggests that the brain makes use of an internal (i.e., generative) model to make inferences about the causes of sensations. This view treats visual sensations as consequent on action (i.e., saccades) and implies that visual percepts must be actively constructed via a sequence of eye movements. Oculomotor control calls on a distributed set of brain sources that includes the dorsal and ventral frontoparietal (attention) networks. We argue that connections from the frontal eye fields to ventral parietal sources represent the mapping from “where”, fixation location to information derived from “what” representations in the ventral visual stream. During scene construction, this mapping must be learned, putatively through changes in the effective connectivity of these synapses. Here, we test the hypothesis that the coupling between the dorsal frontal cortex and the right temporoparietal cortex is modulated during saccadic interrogation of a simple visual scene. Using dynamic causal modeling for magnetoencephalography with (male and female) human participants, we assess the evidence for changes in effective connectivity by comparing models that allow for this modulation with models that do not. We find strong evidence for modulation of connections between the two attention networks; namely, a disinhibition of the ventral network by its dorsal counterpart.

**SIGNIFICANCE STATEMENT** This work draws from recent theoretical accounts of active vision and provides empirical evidence for changes in synaptic efficacy consistent with these computational models. In brief, we used magnetoencephalography in combination with eye-tracking to assess the neural correlates of a form of short-term memory during a dot cancellation task. Using dynamic causal modeling to quantify changes in effective connectivity, we found evidence that the coupling between the dorsal and ventral attention networks changed during the saccadic interrogation of a simple visual scene. Intuitively, this is consistent with the idea that these neuronal connections may encode beliefs about “what I would see if I looked there”, and that this mapping is optimized as new data are obtained with each fixation.

## Introduction

Perception is a fundamentally active process. Although this is true across modalities, it is especially obvious in the visual system, where what we see depends upon where we look (Wurtz et al., 2011; Andreopoulos and Tsotsos, 2013; Ognibene and Baldassarre, 2014; Parr and Friston, 2017a). In this paper, we consider the anatomy that supports decisions about where to look, and the fast plastic changes that underwrite effective saccadic interrogation of a visual scene. We appeal to the metaphor of perception as hypothesis testing (Gregory, 1980), treating each fixation as an experiment to garner new information about states of affairs in the world (Mirza et al., 2016, 2018; Parr and Friston, 2017c). Building upon recent theoretical work (Parr and Friston, 2018), which includes a formal model of the task used here, we hypothesized that the configuration of a visual scene is best represented in terms of expected visual sensations contingent upon a given saccade (“what I would see if I looked there”; Zimmermann and Lappe, 2016). This implies a form of short-term plasticity following each fixation, as the mapping from fixation to observation is optimized.

The purpose of this study is not to evaluate whether we engage in active vision, as there is already substantial evidence in favor of this (Yang et al., 2016; Mirza et al., 2018), but to try to understand how the underlying computations manifest in terms of changes in effective connectivity. Our aim is to establish whether there is neurobiological evidence in favor of optimization of a generative model (Yuille and Kersten, 2006) that represents visual consequences of fixations as a series of eye movements are performed.

In the following, we describe our experimental setup, including our gaze-contingent cancellation task. Through source reconstruction, we demonstrate the engagement of frontal, temporal, and parietal sources, and note the right-lateralization of the temporal component. We then detail the hypothesis in terms of network models or architectures and use DCM to adjudicate between models that do and do not allow for plastic changes in key connections. This model comparison revealed a decrease, from early to late fixations, in the inhibition of neuronal populations in the ventral network by those in the dorsal network.

### Network structure

**Figure 1.**
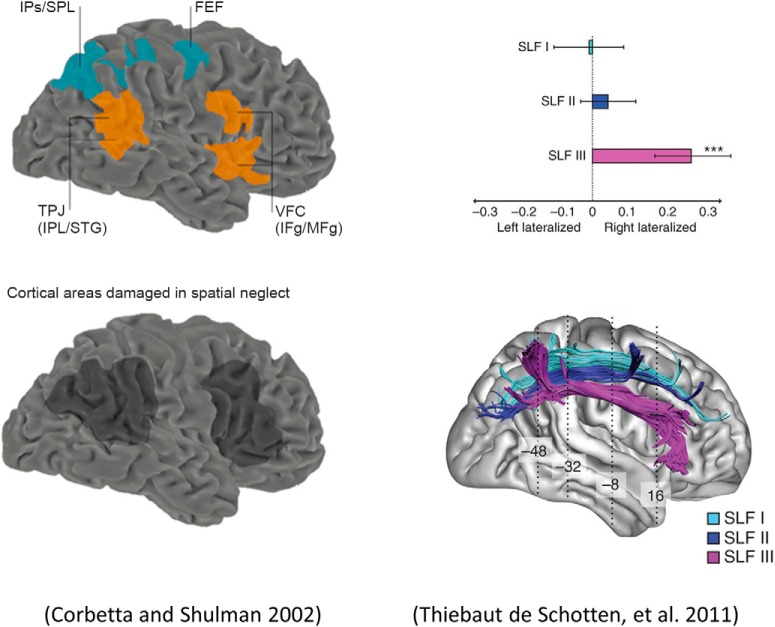
The anatomy of attention. Summary of the functional, neuropsychological, and structural characterizations of attention networks in the brain. Top, Left, The components of the dorsal and ventral frontoparietal attention networks, as derived through functional imaging studies. The dorsal sources (blue) are bilaterally activated during visual attention tasks, whereas the ventral (orange) network is lateralized to the right hemisphere. Bottom, Left, Summarizes lesion studies that demonstrate that lesions to the ventral network in the right hemisphere are associated with visual neglect. Bottom, Right, The three branches of the superior longitudinal fasciculus; a white-matter tract that connects the sources of the attention networks. The plot on the top right indexes the lateralization of these tracts by their relative volumes in each hemisphere. Notably, the third branch, which connects the ventral sources, is significantly right lateralized. Left images are reprinted by permission from Springer Nature: Nature Reviews Neuroscience from (Corbetta and Shulman, 2002), and those on the right reprinted by permission from Springer Nature: Nature Neuroscience from (Thiebaut de Schotten et al., 2011). The material in this figure is not included in the CC BY license for this article. STG, Superior Temporal Gyrus; VFC, Ventral Frontal Cortex; SPL, Superior Parietal Lobule. ***p < 0.001.

The cortical anatomy of oculomotor control has been investigated through functional neuroimaging, neuropsychological, and structural connectivity studies. Figure 1 summarizes how their findings converge upon a system that can be separated into a bilateral dorsal frontoparietal network, and a right lateralized ventral network. In brief, functional imaging experiments (Corbetta and Shulman, 2002; Vossel et al., 2012) during visuospatial tasks reveal activation of the frontal eye fields (FEFs) and the intraparietal sulcus (IPS) in both hemispheres, but greater involvement of the right temporoparietal junction (TPJ) than its contralateral homolog. The volumes of the white-matter tracts connecting the components of the dorsal attention network are comparable, whereas those connecting the ventral network sources are of a significantly greater volume in the right hemisphere (Thiebaut de Schotten et al., 2011). Neuropsychological asymmetries reinforce this network structure, with right hemispheric lesions much more likely than left to give rise to visual neglect (Halligan and Marshall, 1998).

Neglect is a syndrome that manifests as a failure to attend to, or perform exploratory saccades to (Karnath and Rorden, 2012), one side of visual space and (often) appears to be a consequence of a disconnection between the ventral and (right) dorsal networks (Bartolomeo et al., 2007; He et al., 2007). Given the dorsal frontoparietal origins of cortico-collicular axons (Künzle and Akert, 1977; Fries, 1984, 1985; Gaymard et al., 2003), frontal control of eye position (Bruce et al., 1985; Sajad et al., 2015), and the representation of visual stimulus identity in the ventral visual (“what”) stream (Goodale and Milner, 1992; Ungerleider and Haxby, 1994), this is consistent with the idea that the connection between these networks is the neural substrate of an embodied (oculomotor) map of visual space. It is worth noting that the temporoparietal component of the ventral attention network is not within the ventral visual stream. However, it has been associated with target-detection operations (Corbetta and Shulman, 2002; Serences et al., 2005; Chica et al., 2011) that rely upon a simple form of visually derived stimulus identity. Although our focus is in the visual domain, we note that similar networks appear to be involved in auditory attention and neglect (Dietz et al., 2014).

Synthesizing these theoretical and neuroanatomical constructs, we hypothesized that the coupling between the dorsal and ventral attention networks changes with successive fixations in a saccadic task. This hypothesis is based upon the idea that, as an internal model of the task is optimized, the relationship between fixation locations and their visual consequences should become more precise (as demonstrated through simulation in (Parr and Friston, 2018)). If this is the case, this could manifest in one of two ways. The effective connectivity from the temporoparietal cortex to the FEFs could increase over time. Alternatively, plastic changes in connections in the opposite (dorsal-to-ventral) direction could decrease their effective connectivity to relieve descending inhibition of the ventral-to-dorsal projections arising from superficial pyramidal cells. Ultimately, both of these would enhance the influence of ventral parietal over dorsal frontal regions. We used an oculomotor cancellation paradigm, based upon the classic pen-and-paper line cancellation task used to assess visual neglect (Albert, 1973; Fullerton et al., 1986). In this task, patients with neglect tend to cancel (by crossing out) lines on the right side of a piece of paper but miss those on the left. Using magnetoencephalography (MEG) and dynamic causal modeling (DCM) for evoked responses (David et al., 2006) we assessed changes in effective connectivity between dorsal frontal and ventral temporoparietal sources during early and late cancellations (fixations) in healthy participants. Our task involved performing saccades to targets on a screen that, once fixated, changed color and were considered cancelled.

## Materials and Methods

### 

#### Experimental design and statistical analyses

##### Imaging and behavioral task.

We recruited 14 healthy right-handed participants (8 females and 6 males) between the ages of 18 and 35 from the UCL ICN subject pool under minimum risk ethics. Participants were seated in the MEG scanner (whole-head 275-channel axial gradiometer system, 600 samples per second, CTF Omega, VSM MedTech), with a screen ∼64 cm in front of them, showing the stimulus display (size 40 × 29.5 cm). This was presented using Cogent 2000 (developed by the Cogent team at the FIL and the ICN and Cogent Graphics developed by John Romaya at the LON at the Wellcome Department of Imaging Neuroscience).

**Figure 2.**
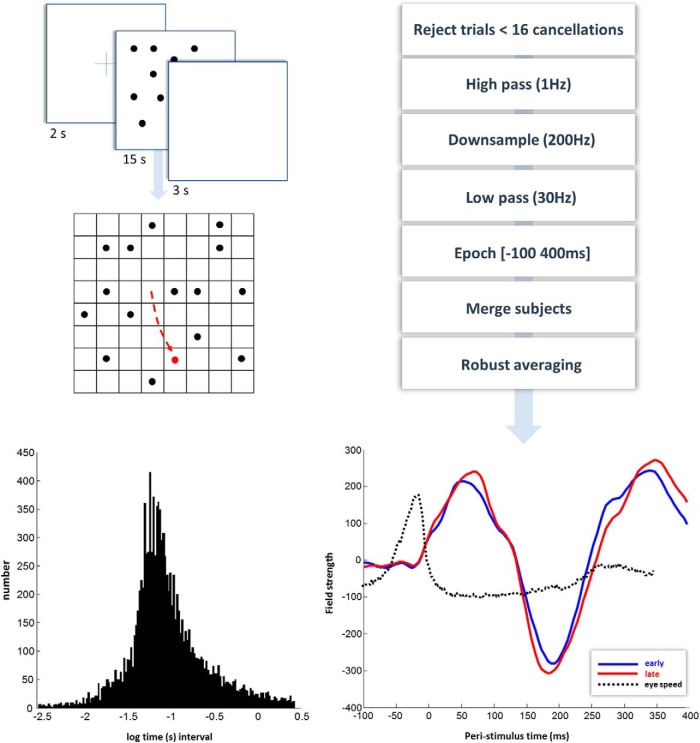
Oculomotor cancellation task and preprocessing. Top, Left, The sequence of events for a given trial. First, a fixation cross is presented for 2 s. After this, a display with 16 black dots is randomly generated and presented for 15 s. This is followed by a blank screen for 3 s. The dots were placed within an 8 × 8 grid (not visible to the participants), as shown at the bottom. When the dots were visible on screen, we tracked the eyes of the participant. Whenever their gaze entered a square containing a black dot, this changed from black to red and remained red for the rest of the trial. Participants were instructed to look at the black dots, and to avoid looking at red dots. Events were defined as the time at which the eye crossed into the square, causing a change in color (i.e., a cancellation). There were 15 of these trials per block, with 6 blocks per participant. The bottom left plot shows a histogram of the time intervals between saccadic dot cancellations, to give a sense of the latency between saccades. These latencies are reported using a (natural) logarithmic time scale (with time in seconds) over the first 2.5 SD above and below the mean. The mean here is −1.0597, corresponding to ∼3 cancellations per second (consistent with the 3–4 Hz frequency of saccadic sampling; Hoffman et al., 2013). Right, The sequence of preprocessing steps used and the first principal component of the ensuing evoked response. The evoked response to early cancellations is averaged from 6738 events, and the response to late cancellations from 6571. Superimposed upon this is a trace of the eye speed in peristimulus time in arbitrary units. This is aligned so that zero corresponds to the average speed during the time in which the fixation cross was present.

The sequence of stimuli is illustrated in Figure 2. Following a fixation cross, a set of 16 black dots appeared on the screen, simultaneously, in pseudorandom (using the MATLAB random number generator) locations. When a dot was fixated, it changed from black to red (i.e., was “cancelled”). Participants were asked to look at the black dots, but to avoid looking at the red dots. We tracked the eyes of the participants while the dots were on screen using an SR Research eye-tracker (Eyelink 1000, operated using Psychtoolbox) sampling at a frequency of 1 kHz. We divided the cancellation events into two categories: early (first 8) and late (last 8).

Although almost all perceptual tasks call upon some sort of engagement with the sensorium, this task emphasizes the active nature of visual processing through making the visual element of the task as simple as possible. This still calls upon optimization of beliefs under an internal model, as formalized by Parr and Friston (2018). As outlined above, this has some validity in relation to disorders in which active vision is impaired. However, it is worth noting that other approaches to studying these processes, particularly those that focus on behavioral (as opposed to neurophysiological) measures (Yang et al., 2016; Mirza et al., 2018), make use of more complicated visual stimuli, so that different saccades afford different levels of information gain about a particular scene category.

Our preprocessing steps (using SPM 12, http://www.fil.ion.ucl.ac.uk/spm/software/spm12/) are specified in Figure 2. As participants generally had no trouble in cancelling all 16 dots, we rejected all trials for which they were unable to do so (assuming these were due to eye-tracker calibration errors). We merged the epoched data from all participants, and averaged the epochs corresponding to the first eight, and the last eight, cancellations over all participants to create a grand average. This meant we averaged over fixations preceded by saccades from all possible directions, ensuring any directional eye movement induced artifacts following cancellation were averaged away. Using robust averaging provides an additional protection against artifactual signals, as this iterative procedure rejects those trials that deviate markedly from the mean response. The average eye-speed is shown in Figure 2 (black dotted line) to illustrate that it falls to its minimum at about the same time as the target is cancelled. The first principal component, across spatial channels, of the averaged evoked response (to a cancellation) in each condition is shown on the same plot. To further interrogate the changes in effective connectivity, we additionally constructed grand averaged responses to each of the 16 cancellations in a trial. These were used for the more detailed model of (parametric) time-dependent responses described in the results section.

##### Source reconstruction.

**Figure 3.**
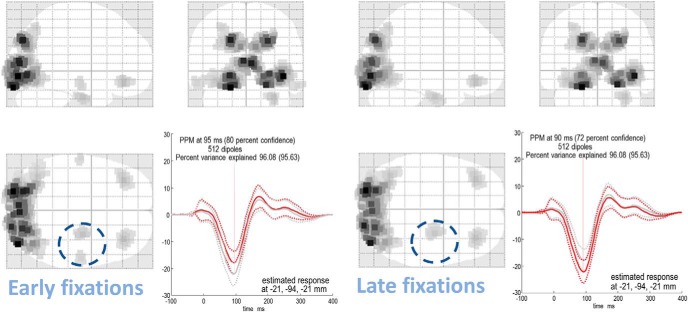
Source reconstruction with multiple sparse priors. These images show the Bayes optimal source reconstruction under multiple sparse priors (and following application of a temporal Hanning window) for the first eight cancellations (left) and the second eight cancellations (right) in a trial. This reveals a set of symmetrical sources in both the frontal and posterior cortical sources, with a right lateralized temporal component. The striking asymmetry of these temporal sources (dashed circles) is encouraging, considering the known rightward lateralization of the ventral attention network. Although we might expect the frontal sources to be more dorsal, this may reflect the ill-posed nature of MEG source localization; there are many possible combinations of sources in 3D space that could give rise to the same pattern of activation over the 2D sensory array. The estimated responses show the greatest amplitude at ∼100 ms. In the left plot (showing the maximal response for the first condition), the red lines indicate the reconstructed activity from the early cancellations and gray from the late cancellations. In the right plot (maximal response for the second condition), red is late and gray is early. Bayesian credible intervals are shown as dotted lines for each response. The confidence associated with the posterior probability maps (PPM; Friston and Penny, 2003), in addition to the variance explained, are included in the top left of each plot, and the location at which the response is estimated is given at the bottom right.

In Figure 3, we show the reconstructed source activity obtained using multiple sparse priors (Friston et al., 2008). This scheme tries to infer the sources in the brain that generated the data measured at the sensors. There are an infinite number of possible solutions to this problem, but Bayesian methods attempt to find the simplest of these. Our results, using standard settings (Litvak et al., 2011), show a relatively symmetrical distribution of frontal and posterior cortical sources, and a right lateralized (asymmetrical) temporal component. While the inferred locations are more ventromedial than we might expect, based upon Figure 1, (likely because of the ill posed nature of the MEG inverse problem). It is encouraging that we can recover sources that are broadly consistent with the known anatomy, and lateralization, of the attention networks (Corbetta and Shulman, 2002) from these data.

##### Dynamic causal modeling.

DCM tries to explain measured electrophysiological data in terms of underlying neuronal (i.e., source) activity (Friston et al., 2003). This rests upon optimizing the model evidence (or free energy) for a biophysically plausible neural mass model. The (log) evidence that data **y** affords a model *m* is as follows:

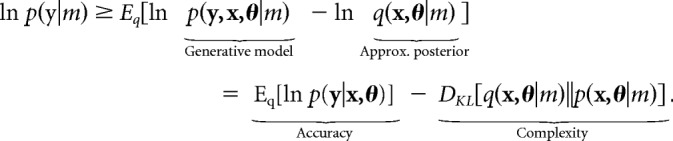
 DCM makes use of a variational Laplace procedure (Friston et al., 2007) to optimize beliefs (*q*) about neuronal activity (**x**) and the parameters (θ) that determine this activity (e.g., connection strengths) and the (likelihood) mapping (e.g., lead field) from **x** to **y**. The lead field matrix maps source activity to the measured sensor data on the scalp (Kiebel et al., 2006). In maximizing model evidence, DCM finds the most accurate explanation for the data that complies with Occam's principle; i.e., is minimally complex (as measured by the KL divergence between posteriors and priors). By comparing different generative models, we can test hypotheses about biologically grounded model parameters; here, condition-specific changes in connectivity under a particular network architecture.

**Figure 4.**
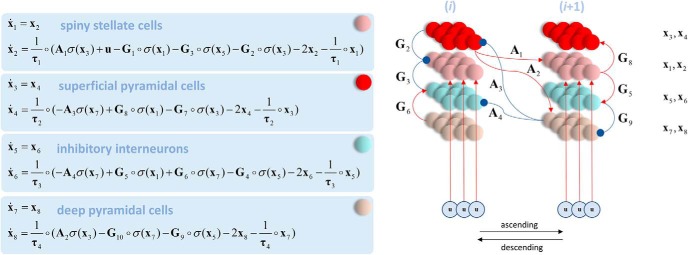
The canonical microcircuit. The equations on the left of this schematic describe the dynamics of the generative model that underwrites the dynamic causal modeling in this paper. The **x** vectors represent population-specific voltage (odd subscripts) and conductance (even subscripts). Each element of the **x** vectors represents a distinct cortical source. The notation **a ○ b** means the element-wise product of **a** and **b**. The matrix **A** determines extrinsic (between-source) connectivity (here illustrated as connections between a lower source *i* and a higher source *i*+1), whereas **G** determines the intrinsic (within-source) connectivity. Subscripts for these matrices indicate mappings between specific cell populations. For example, **A**_1_ describes ascending connections from superficial pyramidal cells (source *i*) to spiny stellate cells (source *i*+1), whereas **A**_3_ describes descending connections from deep pyramidal cells (source *i*+1) to superficial pyramidal cells (source *i*). Experimental inputs, in our case, the cancellation of the target on fixation, are specified by **u**. Right, The neuronal message passing implied by these equations. Red arrows indicate excitatory connections and blue inhibitory. Superficial pyramidal cells give rise to ascending connections that target spiny stellate and deep pyramidal cells in a higher cortical source. Descending connections arise from deep pyramidal cells that target superficial pyramidal cells and inhibitory interneurons.

The generative model we used is the canonical microcircuit model (Bastos et al., 2012; Moran et al., 2013), which incorporates four distinct neuronal populations (Fig. 4). These are spiny stellate cells, superficial and deep pyramidal cells, and inhibitory interneurons. The connections associated with each of these populations conforms to known patterns of laminar-specific connectivity in the cerebral cortex (Zeki and Shipp, 1988; Felleman and Van Essen, 1991; Shipp, 2007), allowing us to distinguish between ascending and descending extrinsic (i.e., between source) connections. This accounts for the prior probability density *p*(**x,θ**|*m*) that, supplemented with a lead-field provides a likelihood *p*(**y**|**x,θ**) and completes the forward or generative model.

**Figure 5.**
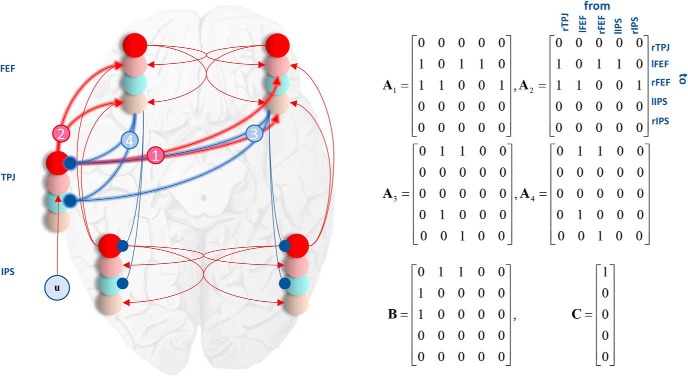
Network architecture. This schematic illustrates the form of the network model we used to test our hypothesis. The dorsal network is present bilaterally (FEF and IPS) and is connected to the ventral network, represented by the TPJ, on the right. The TPJ receives input as it sits lower in the visual hierarchy than the FEF (Felleman and Van Essen, 1991). Our hypothesis concerns the (highlighted) connections between the two networks. We compared models that allowed for changes or visual search-dependent plasticity in connections from the TPJ to left FEF (1), from the TPJ to right FEF (2), from the left FEF to TPJ (3), from the right FEF to TPJ (4), and every combination of the above. The matrices on the right illustrate the specification of these connections. The **A** matrices are the same as those in Figure 4 and represent extrinsic connections between sources (with subscripts indicating which specific cell populations in those sources). **B** specifies the connections that can change between the early and late cancellations and **C** specifies which sources receive visual (i.e., geniculate) input. To ensure that the signs of the **A** (and **C**) connections do not change during estimation, their logarithms are treated as normally distributed random variables. This ensures an excitatory connection cannot become an inhibitory connection and vice versa.

As we were interested in changes in the coupling of the dorsal and ventral attention networks, we specified our generative model as in Figure 5; incorporating the bilateral dorsal network and the right lateralized temporoparietal contribution to the ventral network (consistent with the source reconstruction above). The connections between the right TPJ (rTPJ) and the left FEF probably involve an intermediate thalamic relay (Guillery and Sherman, 2002; Halassa and Kastner, 2017), but this was omitted for simplicity. Our hypothesis was that the connections between the rTPJ and each FEF would change between early and late target cancellations (Parr and Friston, 2018). Figure 5 highlights these ascending and descending connections. After fitting the full model (with modulation of all four connections) to our empirical data, we used Bayesian model reduction (Friston et al., 2017) to evaluate the evidence for models with every combination of these condition-specific effects (early vs late) enabled or set, a priori, to zero.

## Results

**Figure 6.**
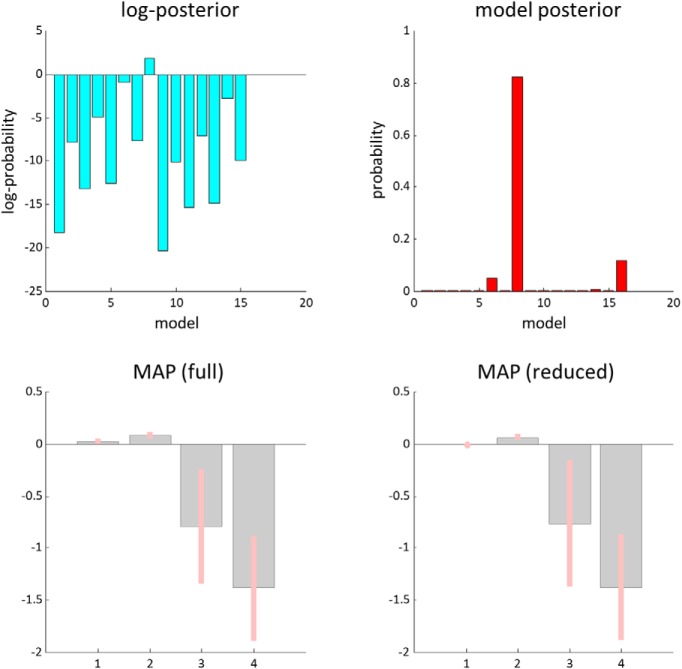
Model comparison and Bayesian model averaging. This figure shows the results of comparing models with different combinations of condition-specific effects on the forward and backward connections between the right TPJ and the FEFs. We performed this comparison using Bayesian model reduction (Friston et al., 2017), which involves fitting a full model that allows all four connections to change and analytically evaluating the evidence for models with combinations of these changes switched off. The top plots show the log posterior probabilities associated with each model, and the posterior probabilities. The winning model (number 8) allows for modulation in Connections 2, 3, and 4 (Fig. 5). The bottom plots show that, for the later fixations, there is a modest increase in the effective connectivity in Connection 2, but a decrease in 3 and 4. These values correspond to log scaling parameters, such that a value of zero means no change. The bottom left plot shows these parameter (maximum a posteriori) estimates for the full model (that allows for all connections). The bottom right plot shows the Bayesian model average of these estimates (weighted by the probability of each reduced model to account for uncertainty over models). Bayesian 90% credible intervals are shown as pink bars.

Figure 6 reports the results of a model comparison between 16 (2^4^) models that allowed for different patterns of search-dependent changes in the forward and backward connections between each FEF and the rTPJ. Given our grand average data, Model 8 has a posterior probability of 0.827. This model allows for changes in backward connections, and the forward connection from rTPJ to the right FEF, but not to the left FEF. This provides evidence in favor of changes in the efficacy of dorsal-ventral connections. Acknowledging that other models, although improbable, were found to be plausible, we averaged our results across models, weighting each model by its posterior probability. Following this Bayesian model averaging, we still found striking changes in the backward connections, which show a decrease in effective connectivity for late compared with early cancellations. As backward connections are (net) inhibitory, this corresponds to a disinhibition of the superficial pyramidal cells, the origin of ascending connections, in the TPJ. In other words, the effective connectivity during the later stages of the trial changed, compared with that during the first few cancellations, to relieve the inhibitory effect of the dorsal attention network on the source of its input from the ventral network.

**Figure 7.**
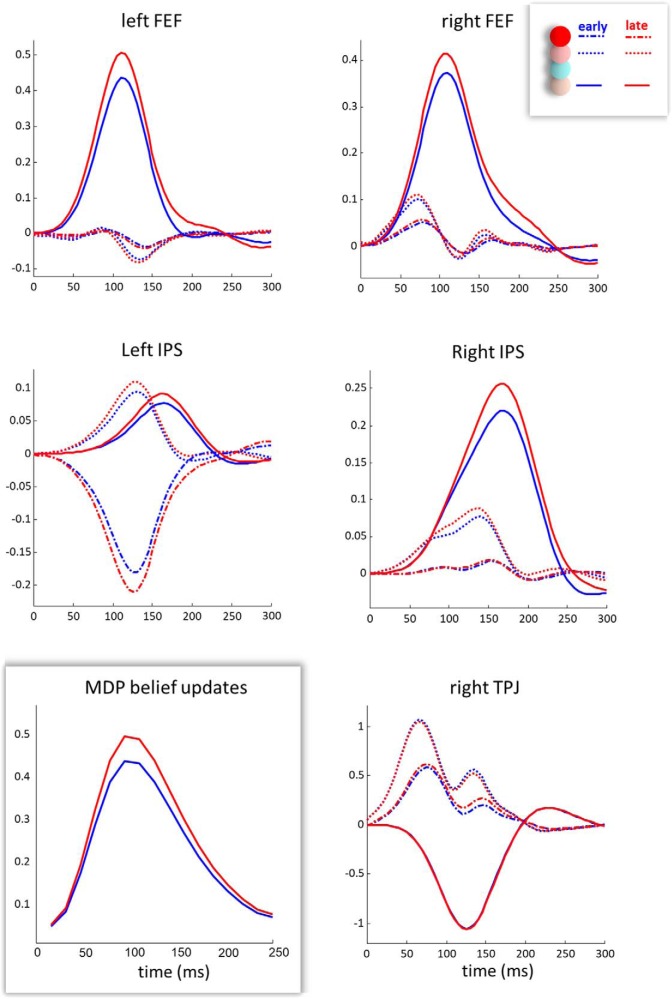
Estimated neuronal activity. These plots show the estimated activity in each excitatory cell population. Dashed lines indicate the superficial pyramidal cells that give rise to ascending connections and are inhibited by higher cortical sources. Ascending connections target the spiny stellate cells (dotted lines), and the deep pyramidal cells (unbroken lines). The latter give rise to descending connections. The activity here is shown for early (blue) and late (red) cancellations, for each of the cortical areas shown in Figure 5. The bottom left plot (highlighted) shows the simulated evoked responses obtained from the Markov decision-process model described by Parr and Friston (2017b), drawing from the process theory associated with active inference (Friston et al., 2016). It is computed by taking the absolute rate of change of the sufficient statistics of posterior beliefs about the current fixation location, summed over spatial scales (please see the discussion for details). Whereas the *y*-axis here is arbitrary, the *x*-axis extends to 250 ms, consistent with the theta frequency of saccadic eye movements. There is a striking resemblance between the simulated rate of belief updating and the FEF neuronal activity estimated from our empirical data.

The effects of this disinhibition can be seen in the reconstructed neuronal activities shown in Figure 7. During later cancellations, the activity of the superficial pyramidal cells in the rTPJ has a greater amplitude than evoked during earlier fixations. Figure 5 shows that this is the population inhibited by the descending connections (labeled 3 and 4). These are the connections that show the greatest change (both relative and absolute, despite being slightly weaker at baseline than the forward connections). Although the change in ascending synapses is small or absent, the increase in activity in these forward projecting TPJ cells has driven an increase in the amplitude of responses in all populations in each FEF. The most dramatic effect is in the deep pyramidal layer, which receives direct input from the superficial TPJ cells. Figure 7 additionally shows the resemblance between the activity in deep pyramidal cells in FEF and the simulated rate of belief updating obtained under a Markov decision-process model of the same behavioral task: for details, see Parr and Friston (2018). This model represents a formalization of the ideas raised in the introduction; namely, that representations of visual space depend upon beliefs about the sensory consequences of actions. In brief, the differences in the rate of belief updating from early to late fixations are due to the optimization of the mapping from fixation location to the presence or absence of a target. More precise beliefs later in the task enable faster and more confident belief updates.

**Figure 8.**
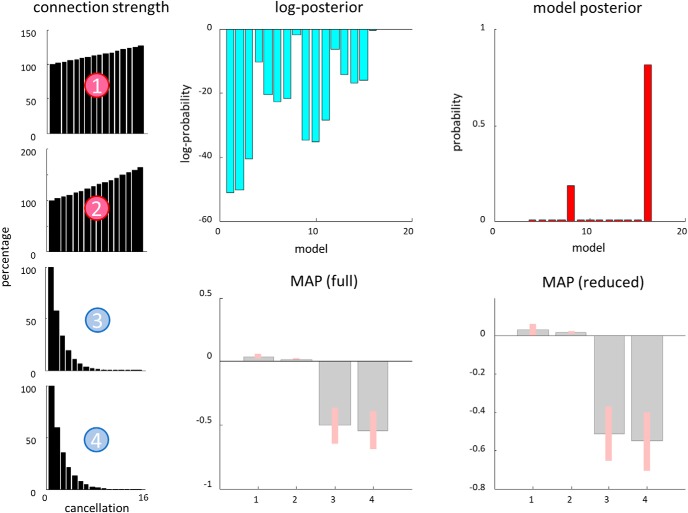
Time-dependency of modulatory changes. The plots on the right are the same as those in Figure 6, but modeling a parametric effect of number of previous cancellations. For this model, in place of the early and late conditions, we treated each sequential cancellation as a separate event. Because the model is parameterized in terms of log-scaling parameters, linear (i.e., [0,1,…,15]) parametric effects of time (number of previous cancellations) correspond to a monoexponential change in coupling [starting from a strength of exp(0), corresponding to 100%]. The two most probable models are the same as in Figure 6, and the overall pattern of changes shown in the MAP estimates is the same (but with some evidence in favor of a small change in Connection 1). The plots on the left show the estimated changes in each connection with successive cancellation events, as a percentage of their initial values. These indicate an increase in the strength of forward excitatory connections over time, and a decrease in backward inhibitory connections.

To explore the changes in coupling demonstrated above in a more parametric way, we inverted a DCM that was identical to that described above, but treated each cancellation as a separate event. This meant that, in place of the relatively coarse division into “early” and “late”, we could test hypotheses about parametric changes in connection strength over 16 sequential cancellations. Figure 8 illustrates a model comparison that tests these hypotheses, endorsing the pattern of changes found in Figure 6. Because of the implicit model of time-dependent effects, this enables us to plot the estimated changes in coupling throughout the trial, as shown in Figure 8. These show a progressive decrease in the strength of inhibitory backward connections, with a modest increase over time in excitatory forward connections.

## Discussion

The results presented here provide evidence in favor of short term plastic changes in the connections between the dorsal and ventral attention networks during the active interrogation of a simple visual scene. This supports an enactive perspective on visual cognition (Hohwy, 2007; Vernon, 2008; Bruineberg, 2017), as it is consistent with the idea that we represent visual sensations as the consequences of action, and that these contingencies may be learned over a short time period. Although these results have interesting implications for active vision, they also constrain the way in which cortical neuronal circuits might implement inferential computations. That the descending connections appear to change the most is consistent with the idea that ascending signals in the brain carry evidence for or against hypotheses represented in higher areas. Although this appears counterintuitive, the evidence afforded to a hypothesis about one variable (e.g., location on a horizontal axis) depends upon beliefs about other variables (e.g., location in the vertical axis). In other words, dorsally represented beliefs about eye position, if represented in any factorized coordinate system, must act to contextualize the ascending signals from the ventral to dorsal network. As this is learned over successive fixations, this contextualization (i.e., interaction between factors) leads to increasingly precise mappings between eye position and its visual consequences; consistent with the disinhibition we observed here. This is analogous to the increase in amplitude of evoked responses following cueing in working memory paradigms (Lenartowicz et al., 2010) that can be reproduced *in silico* by appealing to beliefs about the context of ascending signals (Parr and Friston, 2017b).

An interesting question that arises from this is what type of coordinate system the FEF might use. The argument given above applies regardless of the choice of coordinate system but depends upon there being some factorization (Parr and Friston, 2017a). This factorization could be representation of a horizontal and a vertical axis (McCloskey and Rapp, 2000) or could be closer to a wavelet decomposition, used in computational visual processing (Antonini et al., 1992). The latter separates an image into different spatial scales and resolutions. For example, we might represent which quadrant of space we are looking at and which subquadrant within that quadrant. Either of these systems requires far fewer neurons than we would need if we were to independently represent each location in visual space. This is an important aspect of the normative (active inference) theory on which the simulations in Figure 7 were based. In brief, the sorts of generative models used by the brain to infer the causes of its sensory input are subject to exactly the same imperatives used in Bayesian model comparison; namely, the brain's generative or forward models must provide an accurate account of sensations with the minimum complexity. Reducing the number of parameters via factorization is, in theory, an important aspect of minimizing complexity or redundancy (Barlow, 1961, 1974; Tenenbaum et al., 2011; Friston and Buzsáki, 2016). We used a decomposition of location into quadrants to simulate the belief updating shown in the bottom left of Figure 7, which enables us to reproduce visual neglect at different spatial scales (Parr and Friston, 2018), consistent with neuropsychological observations (Ota et al., 2001; Grimsen et al., 2008; Medina et al., 2009; Verdon et al., 2010).

Visual neglect is increasingly recognized as a disconnection syndrome (He et al., 2007). Specifically, it can arise through damage to the white-matter tracts that link right dorsal frontal sources to ventral temporoparietal areas (Doricchi and Tomaiuolo, 2003; Thiebaut de Schotten et al., 2005). A disconnection of this sort would preclude the changes we have observed in these connections. From the perspective of active inference, this means that saccades to the left side of space represent poor perceptual experiments, as the capacity to learn from them is diminished (Lindley, 1956; MacKay, 1992; Denzler and Brown, 2002; Yang et al., 2016). We have previously argued that syndromes in which active scene construction is impaired, visual neglect being an important example, may result from pathological prior beliefs about these action–sensation mappings (Parr et al., 2018). An inability to change this mapping following observation, perhaps because of white-matter disconnection (Geschwind, 1965; Catani and ffytche, 2005), means that actions that would otherwise engage (and modify) a given connection afford a smaller opportunity for novelty resolution (Parr and Friston, 2018). The failure to update this mapping is consistent with the impairments in spatial working memory that have been elicited in saccadic tasks in neglect patients (Husain et al., 2001). In future work, we aim to follow up this idea by temporarily disrupting changes in these (dorsal-ventral) connections using transcranial magnetic stimulation. We hypothesize that this will induce saccadic scan paths consistent with those observed in visual neglect (Fruhmann Berger et al., 2008; Karnath and Rorden, 2012). Encouragingly, this approach has previously been used to induce other features of visual neglect (Ellison et al., 2004; Platz et al., 2016), including changes in line bisection and visual search performance following stimulation of the right TPJ.

An additional direction for future research concerns the use of more complex visual environments. In this study, we kept the visual stimuli as simple as possible. However, many interesting phenomena in active vision can be elicited using more sophisticated, and often dynamic, manipulations. An advantage to using stationary targets is that they induce scanning saccades as opposed to reactive saccades; of the sort associated with a suddenly appearing target. The former are accompanied by greater involvement of the frontal part of the dorsal network, whereas the latter implicates the parietal part (Pierrot-Deseilligny et al., 1995). Given that our hypothesis concerned the frontal regions of the dorsal network, the use of static targets facilitated the involvement of these regions. However, the inclusion of a second condition in which targets suddenly appeared would help us to further interrogate the respective contributions of the frontal and parietal cortices to these processes. We hope to pursue this in future work.

Specifically, it would be interesting to probe the computational mechanisms that underwrite differences between scanning and reactive saccades for both perception and neurobiological measurements (Zimmermann and Lappe, 2016). This may relate to the time required for belief-updating, which itself is likely to depend upon the sorts of beliefs that are updated. Typically, cortical areas that sit higher in the anatomical hierarchy (Zeki and Shipp, 1988; Felleman and Van Essen, 1991; Shipp, 2007) are thought to represent stimuli that evolve over longer time-periods (Hasson et al., 2008, 2015; Kiebel et al., 2008; Murray et al., 2014), in relation to early sensory cortices. Given that the FEFs are engaged in control of scanning saccades, which occur at ∼3–4 Hz, it is plausible that the time-scale for updating beliefs about “where I am looking” corresponds to this frequency. Speculatively, short-latency reactive saccades may be driven by lower cortical regions (e.g., parietal cortex) that represent the locations of fast-changing stimuli and may not leave enough time for completion of belief updating in frontal areas. As noted by one of our reviewers, this might account for the changes in spatial perception of stationary stimuli that follow adaptive changes in saccadic amplitude, but the absence of this phenomenon when dynamic stimuli induce reactive saccades. This is because, under the view that we represent visual space in terms of the visual consequences of saccades, a failure to complete belief updating, in brain regions representing alternative saccades, may preclude the sort of changes in coupling between frontal and temporoparietal areas observed here. Intuitively, this is sensible when constructing a motor map of visual space: there is little point in including transient stimuli, as they are unlikely to be there on looking back. This idea predicts that there should be a diminished inhibition of return following a reactive, as opposed to a scanning, saccade.

### Conclusion

In this paper, we tested the hypothesis that the coupling between dorsal and ventral frontoparietal networks is altered during visual exploration. To do so, we used dynamic causal modeling based upon a network motivated by pre-existing structural, functional, and neuropsychological data. We found greatest evidence for a model that allowed for modulation in connections from the dorsal to the ventral network. Bayesian modeling averaging revealed a decrease in the effective connectivity of these connections, resulting in a disinhibition of ventral sources by the dorsal attention network. These results are consistent with the idea that the visual data obtained following a saccade drive plastic changes, optimizing beliefs about the sensory consequences of a given saccadic fixation. This has potentially important implications for syndromes in which visual exploration is disrupted; notably, visual neglect. We hope that understanding (and measuring) these changes in effective connectivity in health will yield insights into the pathophysiology of disconnection syndromes.
